# Classic and Non-Classic Effects of the Duration of Supplementation of 25-Hydroxicholecalciferol in Broiler Chicken Diets

**DOI:** 10.3390/ani11102971

**Published:** 2021-10-15

**Authors:** Karen Prokoski, Leticia C. Bittencourt, Levy V. Teixeira, Cristiano Bortoluzzi, Elisangela Vanroo, Sabrina Palma, Jovanir I. M. Fernandes

**Affiliations:** 1 Laboratory of Poultry Experimentation, Federal University of Parana, Palotina 85950-000, Brazil; karenprokoski@yahoo.com.br (K.P.); elizangela.vanroo@gmail.com (E.V.); sabrina.castro@ufpr.br (S.P.); jimfernandes@ufpr.br (J.I.M.F.); 2 DSM Nutritional Products, Sao Paulo 18120-000, Brazil; leticia.cardoso@dsm.com (L.C.B.); cristiano.bortoluzzi@dsm.com (C.B.)

**Keywords:** broiler, breast yield, mTOR, 25(OH)D_3_

## Abstract

**Simple Summary:**

Genetic programs of modern commercial-type broiler chickens are based on growth performance parameters associated with muscular growth, feed efficiency, and meat and breast yield. Therefore, highly bioavailable vitamin D_3_ to sustain optimal muscle and bone development is necessary. In the present study the effect of different durations of supplementing of 25-hydroxycholecalciferol (25(OH)D_3_) in broiler chickens was evaluated. Growth performance response, carcass and cuts yield, bone resistance, plasma concentration of 25(OH)D_3_, and expression of the mTOR gene were evaluated. The use of 25(OH)D_3_ in the diets for broilers during different feeding periods did not influence growth performance but increased the plasma concentration of 25(OH)D_3_, and increased breast yield as the duration of the supplementation was extended. The supplementation of 25(OH)D_3_ increased breast yield and protein deposition in the breast muscle which may have been a result of the epigenetic changes caused by this vitamin D (Vit D) metabolite.

**Abstract:**

The present study aimed to determine the effect of different times of supplementation of 25-hydroxycholecalciferol (25(OH)D_3_) in broiler chickens on the performance, carcass and cuts yield, bone resistance, plasma concentration of 25(OH)D_3_, and expression of the mTOR gene. The treatments were a control diet (CD) supplemented with 3000 IU vitamin D_3_/kg of feed from 1 to 46 d, or the CD + 2760 IU (69 mcg) of 25(OH)D_3_/kg of feed from 1 to 21 d, from 1 to 35 d, or from 1 to 46 d. The period of supplementation of 25(OH)D_3_ did not affect the growth performance of broilers, but the breast meat yield was linearly increased in response to increasing days of supplementation (*p* < 0.05). Birds supplemented with 25(OH)D_3_ at the time of the analysis showed an increase (*p* < 0.05) in the plasma concentration of 25(OH)D_3_ when compared to non-supplemented birds. The mTOR gene expression (*p* < 0.05), and breast protein deposition (*p* < 0.05) presented a quadratic response related to the supplementation period of 25(OH)D_3_. The fat content of the breast linearly decreased (*p* < 0.05) as the period of supplementation was extended. The results also showed a positive linear correlation between mTOR expression and 25(OH)D_3_ plasma levels (r = 0.593; *p* < 0.05).

## 1. Introduction

The Brazilian poultry industry has shown significant growth in the last few decades mainly to meet the internal demand, and to support the high exporting rates observed in the past years [1]. The genetic programs of modern commercial-type broiler chickens are based on growth performance parameters associated mainly with an elevated muscular growth, feed efficiency, and meat and breast yield. However, this high growth rate may increase the needs for more bioavailable nutrients and for higher absorption rates to sustain optimal muscle and bone development, which otherwise would result in the appearance of locomotor problems.

With the objective of reducing losses related to the locomotor system, research has focused on nutritional alternatives to mitigate such problems. Work on vitamin D and its metabolites has been a subject of major interest when studying nutritional-skeletal abnormality interactions [2]. Vitamin D_3_ and its metabolites are of paramount importance to maintain the homeostasis of calcium (Ca) and phosphorus (P) by regulating their intestinal uptake, renal excretion, and bone structure, functions that are classically described in the literature [2,3]. However, vitamin D_3_ is not metabolically active, being necessary to go through two steps of hydroxylation. The first hydroxylation renders 25-Hydroxycholecalciferol (25(OH)D_3_) which is the main metabolite found in the plasma and constitute an important storage of the vitamin. The second hydroxylation results in the production of 1,25-dihydroxycholecalciferol, the biologically active form of vitamin D [3]. Different metabolites of vitamin D (D_3_, 25(OH)D_3_, 1,25(OH_2_)D_3_) are available for animal nutrition, with the objective to supply a more active form of the vitamin, to reduce the energetic expenditure of its metabolism, and to obtain better outcomes, such as performance and meat yield [4,5,6,7].

Several studies have demonstrated that 25(OH)D_3_ has higher metabolic activity and is a more efficient source of vitamin D in diets of broilers when compared to vitamin D_3_. It has been reported that the absorption of 25(OH)D_3_ is 83% faster compared to vitamin D_3_ in broilers [8]. This higher absorption rate may be related to the fact that 25(OH)D_3_ has higher affinity (at least 1000-fold) to intestinal receptors than other vitamin D metabolites [9]. Several studies in poultry have shown a better growth performance and overall bone health when 25(OH)D_3_ was included in the diet [5,6,7,10]. It was observed that 25(OH)D_3_ supplementation was twice as effective as vitamin D_3_ in promoting weight gain and bone resistance in broilers [6] and that it increased bone resistance by 36% in 21-d-old chickens [11] and tended to increase mineral density of femur and tibia in laying hens at late stages of production (>57 weeks) [7] or under height stocking density [12].

These results highlight the classic effects of 25(OH)D_3_ in improving performance and bone quality in poultry. There are questions as to whether the duration of supplementation would influence its non-classical effects in broilers that remain to be answered. To our knowledge, no studies have evaluated times of supplementation of 25(OH)D_3_ in broilers and incorporated the non-classical effects (expression of mTOR gene, carcass yield, and deposition of fat and protein in the breast muscle) in parallel with the classical effects (growth performance and bone quality). Epigenetic changes of genes related to the metabolism and deposition of proteins could explain, in part, the better breast yield observed in birds supplemented with 25(OH)D_3_ [13]. It was demonstrated that 25(OH)D_3_ increased its plasma concentration and protein synthesis in the breast muscle 3-fold and upregulated the protein expression of mTOR [13]. The mTOR pathway controls protein synthesis by phosphorylating translational regulators, which in turn enhances mRNA biogenesis [14]. Nevertheless, 25(OH)D_3_ supplementation improve different aspects of the intestinal physiology, immunology, and microbiology in broilers [11,15,16,17].

Therefore, we hypothesized that a longer period of supplementation of the 25(OH)D_3_ metabolite would maintain its plasma concentration and upregulate the expression of the mTOR gene in the breast muscle, which would be translated into improved growth performance, carcass yield, and bone resistance in broiler chickens. The objective of the present study was to evaluate different durations of supplementation of 25(OH)D_3_ metabolite (Hy-D) in broiler chickens on the performance, carcass and cuts yield, tibia resistance, breast protein deposition and fat content, plasma concentration of 25(OH)D_3_, and expression of the mTOR gene.

## 2. Materials and Methods

### 2.1. Birds, House, and Treatments

A total of 1584 one-day-old RossAP95 male broiler chickens were divided into a completely randomized design with four treatments and nine replicate pens of 44 birds each replicate (each replicate pen had similar initial body weight, with ±5%). Vitamin D was supplied in equal concentrations (3000 IU/kg of feed) as vitamin D_3_ in all the diets. The experiment consisted of four treatments—the control diet (CD) supplemented with 3000 IU vitamin D_3_/kg from 1 to 46 d, and three additional treatments that consisted of different times of supplementation of 25(OH)D_3_/kg of feed, as follows: CD + 2760 IU (69 mcg) of 25(OH)D_3_/kg of feed from 1 to 21 d, from 1 to 35 d, or from 1 to 46 d. When the supplemental period of 25(OH)D_3_ ended, the broilers returned to the control diet (Table 1), except when the supplementation lasted from 1 to 46 d of age.

The diets were based on corn and soybean meal and formulated following the chemical composition of feedstuffs and nutritional recommendations adopted by the regional poultry industry (Palotina, Paraná, Brazil; Table 2). The feeding program was divided into three phases—starter (1 to 21 d), grower (1 to 35 d), and finisher (1 to 46 d). The concentration of vitamin D used in the experimental diets were based on the DSM Vitamin Supplementation guidelines (DSM ROVIMIX Hy-D; DSM Nutritional Products, Basel, Switzerland) for broiler chickens (DSM, 2011). The vitamin D metabolite, 25(OH)D_3_, was included in the vitamin and mineral premix according to the treatment and feeding phase, and its concentration in the diets has been determined and is presented in Table 2.

The birds were vaccinated at the hatchery against Marek’s disease, infectious bursal disease, and infectious bronchitis, and placed into floor pens with re-used litter (4th rearing flock). The temperature in the experimental poultry house was maintained by the use of electric heaters, exhausters, and pad cooling according to each phase of the chickens. The birds received 24 h of light from d 0 to 14, and 16 h of light and 8 h of dark afterwards. The litter management (revolving) was done on d 14 and 28. Water and feed (mash form) were supplied *ad libitum* throughout the study.

### 2.2. Growth Performance and Carcass Yield

The birds and feed were weighed on 7, 21, 35, and 46 d of age to calculate body weight gain (BWG), feed intake (FI), and feed conversion ratio (FCR). The FCR was corrected by the weekly mortality according to Sakomura and Rostagno [19].

On d 46, 12 birds per pen (108 birds/treatment) were randomly selected to determine carcass and cuts yield. After fasting for six hours, the birds were desensitized by electroshock, slaughtered by bleeding through the jugular vein, scalded, plucked, and eviscerated. To calculate the carcass yield, the weight of the hot eviscerated carcass without feet, head, and abdominal fat was expressed relative to the live weight. The cuts yield (whole breast with skin and bones, legs with bones and skin, back and wings with skin) was determined relative to the weight of the hot eviscerated carcass. The whole breast of each bird was deboned to determine the total breast meat, skinless and boneless filet and sassami yields. Wing, medallion, cartilage, and abdominal fat (fat around cloaca, bursa, gizzard, proventriculus, and abdominal muscles) were determined relative to the hot eviscerated carcass.

### 2.3. Plasma Concentration of 25(OH)D_3_

Plasma concentration of 25(OH)D_3_ was determined on d 21, 35 and 46 from two birds per experimental unit (18 birds/treatment). The blood samples, obtained by venipuncture from the brachial vein, were placed into anticoagulant test tubes and immediately centrifuged at 9000 rpm for 60 s; the plasma was stored at −80 °C. Plasma concentration of 25(OH)D_3_ was performed by liquid chromatography coupled to mass spectrometry (LC-MS), according to the methodology described by Weber et al. [20].

### 2.4. Protein Deposition and Fat Content in the Breast Muscle

The deposition of protein in the breast muscle was determined on day 46 24 h after slaughter from six birds per pen (54 birds/treatment), the same birds that were used for the carcass yield analysis. The right pectoralis major muscle (breast filet) was individually ground using a meat grinder (ECCEL-MCIE98), and after homogenization smaller samples were dried under 55 °C for 72 h. After drying, the samples were ground in a knife mill, and the protein analysis was performed by the Kjeldahl method, through acid digestion, followed by distillation and titration.

The fat content was determined by histologic evaluation. For this, samples of the pectoralis major muscle were collected (54 birds per treatment), fixed and paraffin embedded. The slices were longitudinally oriented, eight microns thick and submitted to Masson’s trichrome staining (EP-11-20013 code, Easypath, Erviegas Surgical, Inc, São Paulo, Brazil). The images were captured using a high-resolution Media Cibertecnics PRO SERIES digital camera, coupled to an Olympus Bx 40 microscope, 4× magnification. A computerized IMAGE PROPLUS 5.2 image analyzer (Cibertecnics Media) was used to read the images to measure the content of fat intermingling the muscle bundles in relation to the total area of the captured cut. For such, a color was assigned to the measured structure in order to establish the contrast between the collagen, protein, and fat. The percentage of fat was calculated based on the difference between the total area captured and the percentage of collagen and protein.

### 2.5. Tibia Breaking Strength Analysis

For the analysis of tibia resistance, 54 tibias per treatment from the birds used to evaluate protein and fat content analyses were used. After removal of all adherent tissues of the leg of each bird, the bones were submitted to integrity evaluation. The tibia was weighed, and the length and diameter were measured using a digital paquimeter (mm). The Seedor index [21] was obtained by dividing the bone weight (mg) by its length (mm). The tibia was also submitted to the flexural test (bone strength at break) at a rate of constant deformation for viscoelastic material with PMPA equipment (Stable Micro Systems Physical and Mechanical Properties Analysis System) with cell load of 500 kgf, and head speed of 10 mm/s.

### 2.6. mRNA Expression of the mTOR Gene in the Breast Muscle

The mRNA expression of the mTOR gene was analyzed by real-time PCR. The samples of breast muscle (six per treatment) were collected from the cranial portion of the breast, frozen into liquid nitrogen, and sent for the mTOR gene expression analysis. Briefly, the total mRNA was extracted using the Trizol reagent (Invitrogen, Carlsbad, CA, USA). The RNA pellet was dissolved in RNase-free ultrapure water. The total concentration, quality, and integrity of the mRNA were determined using a spectrophotometer (NanoDrop ND 1000, NanoDrop Technologies, Wilmington, DE, USA). For the cDNA preparation, the SuperScripptTM III First-Strand Synthesis Super Mix and Oligo d(T) kit (Invitrogen Corporation, Carlsbad, CA, USA), and RNAse out (Invitrogen Corporation, Carlsbad, CA, USA) were used for mRNA degradation according to the manufacturer’s instructions. The real-time PCR reactions were performed by using the SYBR GREEN fluorescent dye method (SYBR GREEN PCR Master Mix, Applied Biosystems, Foster, CA, USA). Real-time PCR analyses were performed on the Bio-Rad iQ5™ thermal cycler (Bio-Rad Laboratories, Hercules, CA, USA) using the following cycle parameters: 95 °C for 10 min, 40 cycles of denaturation at 95 °C for 15 s, annealing at 60 °C for 1 min, and melting curve at 95 °C to evaluate the specificity.

The mTOR primer sequences were, forward—TTGGGTTTGCTTTCTGTGGCTGTC, and reverse—ACAGACTTCTGCCTCTTGTGAGCA (Accession number XM_417614.2; 119 bp) [22]. The relative standard-curve method was used to quantify the mRNA concentrations of the mTOR gene in relation to the reference gene (β-actin—forward GCCAACAGAGAGAAGATGAC, and reverse—CACCAGAGTCCATCACAATAC). The mRNA relative abundance was calculated according to the method of Livak and Schmittgen [23].

### 2.7. Statistical Analysis

The data were checked for outliers and then subjected to analysis of normality of studentized errors and homogeneity of variances. Pen and bird were considered as the experimental unit for growth performance and remaining analyses, respectively. Based on these assumptions, ANOVA was performed by using the statistical program SAS 9.0. The means showing significance (*p* ≤ 0.05) and trending (0.05 < *p* ≤ 0.10) treatment differences in the ANOVA were compared by Tukey’s test. Polynomial regression (PROC REG) as a function of the period of 25(OH)D_3_ supplementation, and Pearson correlation analysis (PROC CORR) between the expression of mTOR and plasma concentration of 25(OH)D_3_ were also performed using SAS 9.0 (*p* ≤ 0.05).

## 3. Results

### 3.1. Growth Performance and Carcass and Cuts Yield

The growth performance results from 1 to 7, 1 to 21, 1 to 35, and 1 to 46 d are shown in Table 3. No effect (*p* > 0.05) of the dietary supplementation of 25(OH)D_3_ on the BWG, FI, or FCR was observed in any of the phases evaluated.

**Figure 1:**
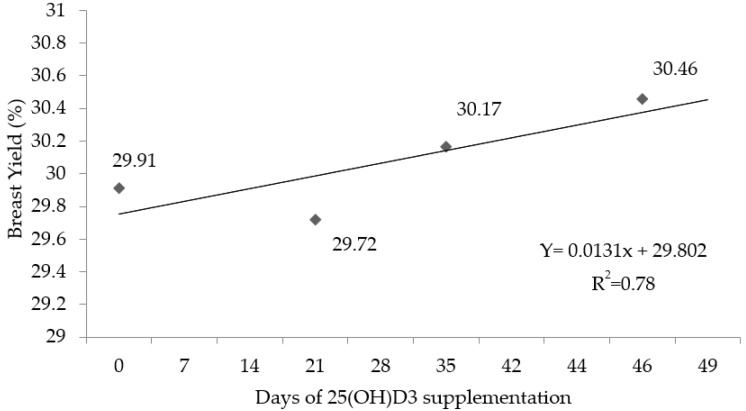
Breast yield (filet + sassami) broiler chickens at 46 d supplemented with vitamin D_3_ from 1 to 46 d or vitamin D_3_ plus 25(OH)D_3_ from 0 to 21, 0 to 35, or 0 to 46 days of age. (*n* = 108 birds/treatment; *p* < 0.05).

The results of carcass yield and cuts are presented in the Table 4. Birds fed diets supplemented with 25(OH)D_3_ from 1 to 46 d had a higher carcass (*p* < 0.05) and breast (*p* < 0.05) yield when compared to birds fed the same diet from 1 to 35 d or from 1 to 21 d, respectively. Additionally, in the regression analysis, an effect (*p* < 0.05) was observed for breast yield as a function of the 25(OH)D_3_ supplementation period (Table 4). The breast yield was linearly increased (*p* < 0.05; Ŷ = 0.0131x + 29.802; Figure 1) by the supplementation period of 25(OH)D_3_, i.e., the longer the birds received of the 25(OH)D_3_ in diet, the higher was the breast yield. The other carcass traits were not influenced (*p* > 0.05) by the supplementation period of 25(OH)D_3_.

### 3.2. Plasma Concentration of 25(OH)D_3_

**Figure 2:**
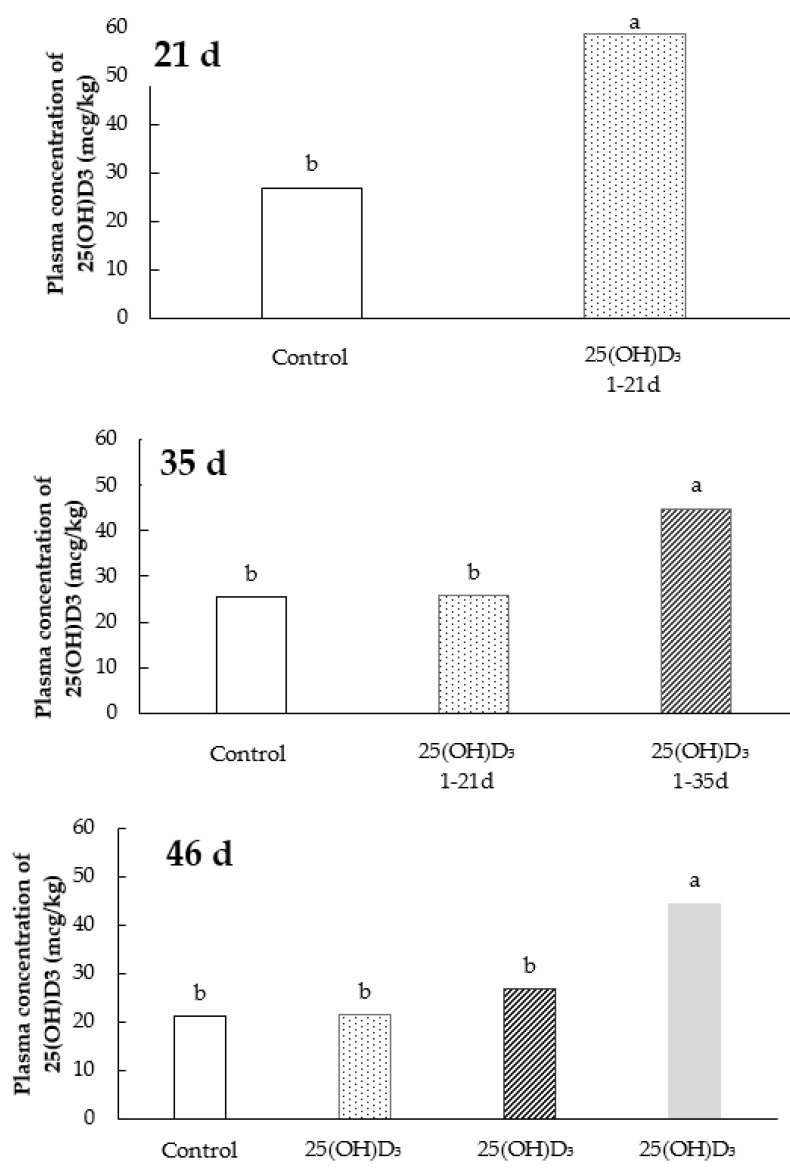
Plasma concentration of 25(OH)D_3_ of broiler chickens at 21, 35, and 46 d supplemented with vitamin D_3_ from 0 to 46 d or vitamin D_3_ plus 25(OH)D_3_ from 0 to 21, 0 to 35, or 0 to 46 days of age. Data are presented as mean ± SEM (*n* = 18/group). Bars with different letters show significant difference between groups (*n* = 18 birds/treatment; *p* < 0.05).

The dietary treatments affected (*p* < 0.05) the plasma concentration of 25(OH)D_3_ in the different periods evaluated (Figure 2). Birds supplemented with 25(OH)D_3_ at the time of the analysis showed an increase of its concentration in the plasma when compared to the non-supplemented birds. It was observed that the maintenance of higher plasma concentration of 25(OH)D_3_ was dependent on its dietary supplementation in association with vitamin D_3_. The vitamin D_3_ supplementation alone was not able to maintain the plasma concentration of 25(OH)D_3_ after the removal of 25(OH)D_3_ metabolite from the diet.

### 3.3. Protein Deposition and Fat Content in the Breast Muscle

**Figure 3:**
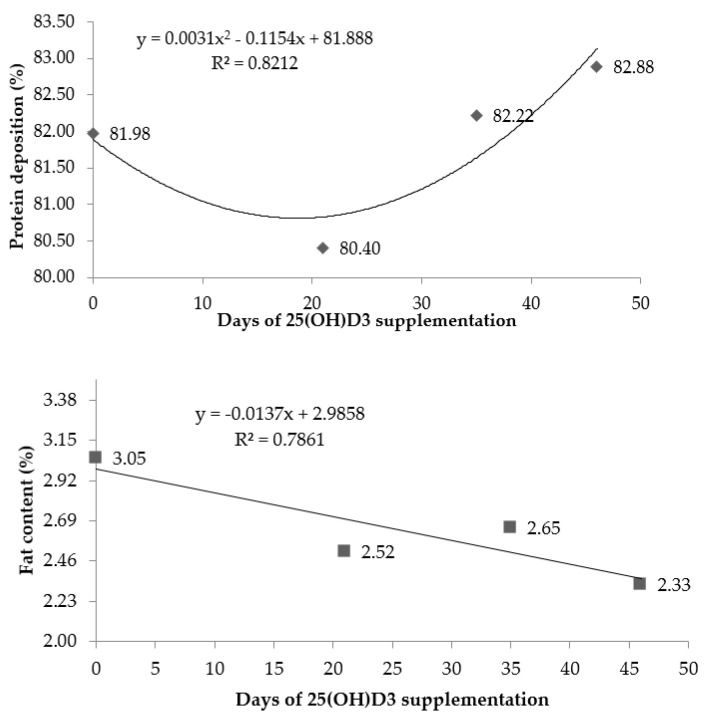
Protein and fat content in the breast muscle of broiler chickens at 46 d supplemented with vitamin D_3_ from 0 to 46 d or vitamin D_3_ plus 25(OH)D_3_ from 0 to 21, 0 to 35, or 0 to 46 days of age. (*n* = 54 birds/treatment).

The results of the protein deposition and fat content in the breast muscle are shown in Figure 3. A quadratic effect was observed (*p* < 0.05; Ŷ = 0.0031x^2^ − 0.1154x + 81.888) on the protein deposition according to the period of 25(OH)D_3_ supplementation, wherein the highest deposition of protein was verified in birds that received 25(OH)D_3_ supplementation during the entire experimental period (1 to 46 d). For the fat content, it was observed a linear effect (*p* < 0.05; Ŷ = −0.0137x + 2.9858), in which the fat content of the breast was reduced as the period of supplementation lasted longer.

### 3.4. Tibia Breaking Strength Analysis

The results of the tibia resistance analysis of broilers performed at 46 d are shown in Table 5. There was a trend (*p* = 0.09) towards increased resistance of the tibia in birds that received the dietary supplementation of 25(OH)D_3_ when compared to the control group of birds, regardless of the duration of supplementation. In addition, a trend (*p* = 0.09) towards higher tibia length was observed in broilers supplemented with 25(OH)D_3_ from 1 to 35 d.

### 3.5. mRNA Expression of the mTOR Gene in the Breast Muscle

**Figure 4:**
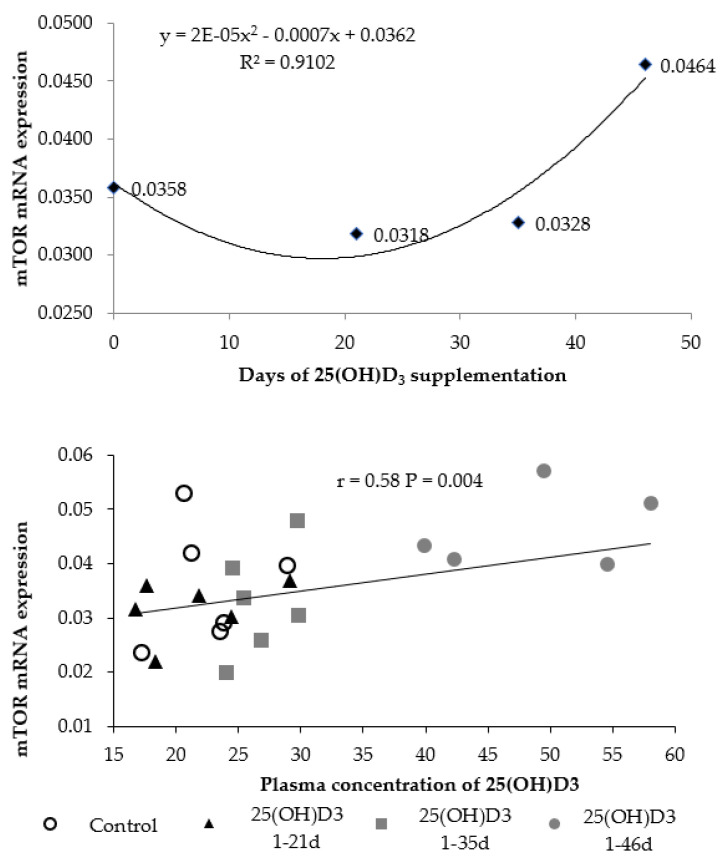
mRNA expression (2^−ΔΔ*C*^_T_) of mTOR protein synthesis initiator gene in the breast muscle and correlation between its expression and the plasma concentration 25(OH)D_3_ of broiler chickens at 46 d supplemented with vitamin D_3_ from 0 to 46 d or vitamin D_3_ plus 25(OH)D_3_ from 0 to 21, 0 to 35, or 0 to 46 days of age. (*n* = 6 birds/treatment for mTOR gene expression and *n* = 18 birds/treatment for plasma concentration of 25(OH)D_3_).

The results of mRNA expression of the mTOR gene in the breast muscle performed at d 46, and its correlation with the plasma concentration of 25(OH)D_3_ are shown in Figure 4. A quadratic increase (*p* < 0.05) in mTOR expression was observed as a function of the timing of 25(OH)D_3_ supplementation, with the higher expression being observed in birds that received 25(OH)D_3_ from 1 to 46 d of age. Additionally, there was a positive correlation (r = 0.58, *p* = 0.004) between the gene expression of mTOR and the plasma concentration of 25(OH)D_3_.

## 4. Discussion

In the present study we evaluated the classical (growth performance, bone resistance, and plasma 25(OH)D_3_ concentration) and non-classical (carcass and cuts yield, fat and protein deposition in the breast, and mTOR gene expression) effects of the 25(OH)D_3_ dietary supplementation in broilers during different periods of supplementation (1 to 21 d, 1 to 35 d, and 1 to 46 d). When the supplemental period of 25(OH)D_3_ ended, the broilers returned to the control diet (Table 1), except when the supplementation lasted from 1 to 46 d of age. We hypothesized that the supplementation of 25(OH)D_3_ throughout the complete production cycle of broiler chickens, would maintain the blood concentration of 25(OH)D3 and upregulate the expression of the mTOR gene in the breast muscle, which would be translated into improved growth performance and carcass characteristics. Overall, the supplementation of 25(OH)D_3_ did not influence growth performance; however, the breast yield was linearly increased by the duration of supplementation, and the tibia resistance tended to increase. The lack of significant result on the tibia resistance may be due to the effect of 25(OH)D_3_ on bone development being more evident around day 21 [11,24], with an enhanced muscle development later in life [13,25].

Supplementing different metabolites in association with a source of vitamin D_3_ is a method of maximizing animal performance since it reduces the energy expenditure with the vitamin D metabolism. It may be possible that if the vitamin D_3_ concentration of the basal diet of the current study was lower, the effects of the addition of 25(OH)D_3_ would have been more pronounced. Moreover, the lack of an immunological stress that would impair the absorption of nutrients, and therefore, the growth performance may explain the absence of significant differences between treatments. Additionally, the adequate levels of nutrients present in the basal diet, mainly Ca and P, may influence the results since the efficacy of vitamin D and its metabolites is dependent upon the availability of nutrients [26,27], or presence of intestinal infection. Indeed, Oikeh et al. [16] showed that coccidiosis infection reduces the plasma concentration of 25(OH)D_3_ at least up to 12 days after the infection. Therefore, due to the constant intestinal challenges faced by the poultry industry more bioactive sources of vitamin D supplemented throughout the life of the birds may be necessary to maximize the intestinal absorption, as observed by Leyva-Jimenez et al. [11].

Similar results to those observed in this study were reported before when evaluating 25(OH)D_3_ for broilers during growth and finisher phases without significant improvement in productive performance [16,28,29,30,31]. However, improved performance with 25(OH)D_3_ supplementation was reported by Fritts and Waldroup [32], wherein 125 IU/kg of 25(OH)D_3_ enhanced the BWG on d 42 to the same level and birds receiving 1000 IU/kg of vitamin D_3_, leading to the conclusion that lower concentrations of vitamin D from 25(OH)D_3_ may be used with greater margin of safety. Furthermore, Sakkas et al. [31] reassessed the effect of higher dietary concentration of vitamin D_3_ and the partial replacement of D_3_ by 25(OH)D_3_ in Ross broilers. There was an improved femur and tibia mineralization when 3000 IU/kg of 25(OH)D_3_ was added to the diet, even though no differences in performance were observed, which partially agrees with our results. Interestingly, the partial substitution of vitamin D_3_ by 25(OH)D_3_ increased the serum concentration of 25(OH)D_3_ only on d 11 and 25 but not on d 39 [31]. On the other hand, Leyva-Jimenez et al. [11] reported that the replacement of 50% of vitamin D_3_ by 25(OH)D_3_ increased body weight, tibia breaking strength, and plasma concentration of 25(OH)D_3_ on d 21 (7 days after a coccidiosis vaccine challenge), compared to vitamin-D_3_-only fed birds.

The elevated 25(OH)D_3_ concentration in the plasma obtained in the present study indicates that its dietary supplementation can increase vitamin D status in broilers. Indeed, we reported an increase of plasma 25(OH)D_3_ by 54.1%, 42.3% and 52.4% on d 21, 35, and 46, respectively, when compared to unsupplemented birds. Similar results have been reported before with higher plasma concentration of 25(OH)D_3_ ranging from 22 to 63% when the partial dietary replacement was adopted [16,25,31], with broader increase being observed in younger birds, and by 126% with total replacement of vitamin D3 by 25(OH)D_3_ [13]. The findings of the present study demonstrated that the longer the dietary supplementation of 25(OH)D_3_ the higher was its plasma concentration. This finding must be emphasized because the breast yield linearly increased as a function of the time of supplementation, most likely due to the higher availability of plasma 25(OH)D_3_ to sustain muscle growth. According to Ovesen et al. [33], in humans, about 80% of the circulating activity of vitamin D is in the form of 25(OH)D_3_. Similarly, in chicks, 25(OH)D_3_ is the major metabolite of vitamin D in the blood [34]. The absorption of 25(OH)D_3_ has been reported to be more efficient than vitamin D_3_ in the small intestine [8]. The faster absorption can be attributed to the biding capacity of 25(OH)D_3_ to proteins in the intestinal cells [35], that have about a 1000-fold greater affinity to 25(OH)D_3_ than to other metabolites of vitamin D [35].

Evidence on the mechanisms by which 25(OH)D_3_ influences muscle hypertrophic growth, composition, and fiber size has been reported [13,25,30]. Hutton et al. [25] observed that feeding broiler chickens with 25(OH)D_3_ increased the status of vitamin D in the birds and increased the number of mitotically active satellite cells in the breast muscle, supporting the fact that the proliferation of muscle fiber and satellite cells is stimulated by circulating levels of 25(OH)D_3_ [25]. Even though the exact mechanism of this response is yet to be elucidated, an increase in the muscle development with the supplementation of 25(OH)D_3_ is expected. In the present study, at d 46, an increase of 1.11% in the carcass yield and 2.62% in the breast yield (filet + sassami) was observed in birds supplemented with 25(OH)D_3_ from 1 to 46 d, compared to those supplemented from 1 to 35 or 1 to 21 d, respectively. These results agree with Vignale et al. [13] wherein the supplementation of 25(OH)D_3_ from 1 to 42 d, but not from 1 to 21 d, increased breast yield. The increase in breast yield without changing feed intake and body weight observed in this study indicates that 25(OH)D_3_ can affect energy use as reported by Vignale et al. [13]. Interestingly, besides increasing protein deposition, the time of supplementation of 25(OH)D_3_ linearly reduced the fat content in the breast tissue likely due to the potent effect of 25(OH)D_3_ on muscle gene expression which may indirectly reduce the fat deposition in different tissues, another non-classical effect of this metabolite [36].

Improvement of vitamin D status in metabolism results in changes in muscle activity [14]. In order to define the mechanism by which 25(OH)D_3_ benefits muscle growth in broilers, we also evaluated the mTOR signaling pathway. The mTOR pathway is largely involved in the regulation of protein synthesis [37] and regulates growth and homeostasis of an organism [14]. Vignale et al. [13] showed that the use of dietary 25(OH)D_3_ enhanced breast meat production and led to a 3-fold increase of fractional protein synthesis rate compared to birds supplemented with vitamin D_3_. Molecular analyses revealed that the breast muscle development of chickens fed 25(OH)D_3_ for 42 d is probably mediated by the mTOR-S6K pathway. In the current study, the supplementation of 25(OH)D_3_ throughout the life of the birds upregulated the expression of mTOR in the breast muscle as measured on d 46. Additionally, the correlation analysis showed that the expression of this gene was positively correlated with plasma 25(OH)D_3_, which helps to explain the observed higher breast yield and protein deposition in the breast muscle.

## 5. Conclusions

In conclusion, the use of 25(OH)D_3_ in the diets for broilers during different feeding periods did not influence growth performance but increased the plasma concentration of 25(OH)D_3_ as the duration of supplementation was extended, and linearly increased breast yield as a function of the timing of supplementation. The supplementation of 25(OH)D_3_ increased breast yield and protein deposition and reduced the fat content in the breast muscle which may be a result of the upregulation of the mTOR gene.

## Figures and Tables

**Table 1:** Experimental treatments.

Treatments	Vitamin D	25(OH)D_3_ ^1^	Total Vitamin D Activity	Period
	(IU/kg)	(IU/kg)		(Days)
**T1**	3000	-	3000	0–46
**T2**	3000	2760	5760	0–21
	3000	-	3000	22–46
**T3**	3000	2760	5760	0–35
	3000	-	3000	36–46
**T4**	3000	2760	5760	0–46

^1^ 25(OH)D_3_ was included in the vitamin and mineral premix according to the treatment and feeding phase, and its concentration in the diets has been determined and is presented in Table 2. (1 mcg = 40 IU).

**Table 2:** Nutritional composition of the experimental diets.

Ingredients, %	Starter	Grower	Finisher
Corn 7.5%	60.17	63.85	69.06
Soybean meal 46.7%	30.90	26.80	22.30
Meat bone meal 48.5%	4.500	3.600	2.900
Soybean oil	2.100	3.500	3.600
Limestone	0.580	0.560	0.560
Salt	0.200	0.190	0.200
Sodium bicarbonate	0.290	0.270	0.240
Vitamin and mineral premix ^1^	0.300	0.300	0.300
Choline 60%	0.096	0.074	0.060
DL-Methionine 98%	0.369	0.352	0.320
L-Lysine 50.7%	0.292	0.320	0.322
L-Threonine 98%	0.099	0.100	0.082
L-Valine 96.5%	0.046	0.071	0.060
Salinomycin 12%	-	0.055	-
Nicarbazin + Narasin 80/80	0.05	-	-
Calculated nutritional composition			
CP, %	22.00	20.00	18.00
ME, Kcal/kg	3050	3180	3250
Fat, %	5.233	6.555	6.698
CF, %	2.359	2.210	2.061
Calcium, %	0.950	0.840	0.760
AvP, %	0.470	0.420	0.380
Dig. Lys., %	1.256	1.160	1.040
Dig. AAS,%	0.967	0.906	0.832
Dig. Thr, %	0.829	0.767	0.687
Dig. Trp, %	0.229	0.205	0.179
Dig. Val, %	0.954	0.894	0.801
Na, %	0.200	0.190	0.180
Cl, %	0.240	0.240	0.240
Analyzed 25(OH)D_3_, mcg ^2^	67.9	63.4	63.6

CP: crude protein; ME: metabolizable energy; CF: crude fiber; AvP: available phosphorus. ^1^ Starter feed (provided per kg of feed): Vit A: 12,000 IU; Vit D_3_: 3000 IU; Vit E: 70 mg; Vit K3: 4.50 mg; Vit B1: 2.6 mg; Vit B2: 7.5 mg; Vit B6: 4.5 mg; Vit B12: 0.025 mg; niacin: 50 mg; pantothenic acid: 19 mg; folic acid: 1.9 mg; biotin: 0.18 mg; Se: 0.3 mg; Fe: 0.3 g; Cu: 0.06 g; Mn: 0.39 g; Zn: 0.39 mg; I: 0.006 mg; phytase: 1000 FYT; Rovimix HyD (25(OH)D_3_): 2760 IU (69 mcg). Grower feed (provided per kg of feed): Vit A: 9000 IU; Vit D_3_: 3000 IU; Vit E: 50 mg; Vit K3: 3.75 mg; Vit B1: 2.1 mg; Vit B2: 6 mg; Vit B6: 3.5 mg; Vit B12: 0.02 mg; niacin: 40 mg; pantothenic acid: 15 mg; folic acid: 1.5 mg; biotin: 0.15 mg; Se: 0.3 mg; Fe: 0.3 g; Cu: 0.06 g; Mn: 0.39 g; Zn: 0.39 mg; I: 0.006 mg; phytase: 1000 FYT; Rovimix HyD (25(OH)D_3_): 2760 IU (69 mcg). Finisher feed (provided per kg of feed): Vit A: 7000 IU; Vit D_3_: 3000 IU; Vit E: 35 mg; Vit K3: 2.75 mg; Vit B1: 1.75 mg; Vit B2: 5 mg; Vit B6: 2.5 mg; Vit B12: 0.015 mg; niacin: 30 mg; pantothenic acid: 11 mg; folic acid: 1.1 mg; biotin: 0.10 mg; Se: 0.3 mg; Fe: 0.3 g; Cu: 0.06 g; Mn: 0.39 g; Zn: 0.39 mg; I: 0.006 mg; phytase: 1000 FYT; Rovimix HyD (25(OH)D_3_): 2760 IU (69 mcg). ^2^ Analyzed at the DSM Laboratory (Basel, Switzerland), following Schadt et al. [18].

**Table 3:** Productive performance of broiler chickens supplemented with vitamin D_3_ from 1 to 46 d or vitamin D_3_ plus 25(OH)D_3_ from 0 to 21, 0 to 35, or 0 to 46 days of age.

Treatment	BWG, g	FI, g	FCR
0 to 7 d			
Control ^1^	134.1	161.8	1.208
25(OH)D_3_	134.8	161.4	1.202
CV, %	4.51	3.55	6.28
*p* value	0.75	0.86	0.80
0 to 21 d			
Control ^1^	975	1288	1.322
25(OH)D_3_	979	1284	1.312
CV, %	2.53	2.48	2.36
*p* value	0.63	0.80	0.47
0 to 35 d			
Control ^1^	2395	3461	1.446
25(OH)D_3_ 1–21 d ^2^	2384	3446	1.446
25(OH)D_3_ 1–35 d ^3^	2390	3409	1.427
CV, %	3.65	3.28	1.62
*p* value	0.96	0.48	0.21
0 to 46 d			
Control ^1^	3340	5365	1.606
25(OH)D_3_ 1–21 d ^2^	3314	5300	1.602
25(OH)D_3_ 1–35 d ^3^	3360	5283	1.574
25(OH)D_3_ 1–46 d ^4^	3264	5186	1.590
CV, %	6.23	5.75	2.68
*p* value	0.79	0.66	0.45
Regression	Ns	Ns	Ns

BWG: body weight gain; FI: feed intake; FCR: feed conversion ratio; ¹ control diet (CD): 3000 IU vitamin D_3_; ² CD + 2760 IU/kg 25(OH)D_3_ fed from 0 to 21 days; ³ CD + 2760 IU/kg 25(OH)D_3_ fed from 0 to 35 days; ^4^ CD + 2760 IU/kg 25(OH)D_3_ from 0 to 46 days; CV: coefficient of variation; Ns: not significant. (*n* = 9 pens/treatment; *p* < 0.05).

**Table 4:** Carcass and cuts yield, and abdominal fat deposition of broiler chickens supplemented with vitamin D_3_ from 1 to 46 d or vitamin D_3_ plus 25(OH)D_3_ from 0 to 21, 0 to 35, or 0 to 46 days of age.

Characteristics	Control ^1^	25(OH)D_3_ ²	25(OH)D_3_ ³	25(OH)D_3_ ^4^	CV, %	*p* Value	Regression
		1 to 21 Days	1 to 35 Days	1 to 46 Days			
Carcass, %	80.6 ^a,b^	80.7 ^a,b^	80.2 ^b^	81.1 ^a^	2.8	0.04	Ns
Breast (filet + sassami), %	29.9 ^a,b^	29.7 ^b^	30.2 ^a,b^	30.5 ^a^	6.6	0.04	Linear
Filet, %	24.7	24.7	25.1	25.2	7.1	0.08	Ns
Sassami, %	5.2	5.1	5.1	5.2	10.2	0.07	Ns
Legs, %	31.6	31.7	31.1	31.3	6.2	0.09	Ns
Wings, %	9.9	9.7	9.6	9.6	10.3	0.17	Ns
Back, %	22.3	22.3	22.2	21.9	7.1	0.38	Ns
Medallion, %	1.9	1.9	1.9	1.9	14.7	0.54	Ns
Skin, %	2.3	2.3	2.3	2.3	16.8	0.76	Ns
Breast flaps, %	0.7	0.7	0.7	0.7	35.6	0.97	Ns
Fat, %	0.9	1.9	1.9	1.8	34.8	0.71	Ns
Breast cartilage, %	0.3	0.3	0.3	0.3	26.6	0.53	Ns

¹ Control diet (CD): 3000 IU vitamin D_3_; ² CD + 2760 IU/kg 25(OH)D_3_ fed from 0 to 21 days; ³ CD + 2760 IU/kg 25(OH)D_3_ fed from 0 to 35 days; ^4^ CD + 2760 IU/kg 25(OH)D_3_ from 0 to 46 days; CV: coefficient of variation; Ns: not significant. ^a,b^ Means followed by different letters in the same row differ from each (*n* = 108 birds/treatment; *p* < 0.05).

**Table 5:** Resistance and measurements of tibia at 46 d of broiler chickens supplemented with vitamin D_3_ from 0 to 46 d or vitamin D_3_ plus 25(OH)D_3_ from 0 to 21, 0 to 35, or 0 to 46 days of age.

	Control ^1^	25(OH)D_3_ ²	25(OH)D_3_ ³	25(OH)D_3_ ^4^	CV, %	*p* Value	Regression
		1 to 21 d	1 to 35 d	1 to 46 d			
Resistance, kg	43.8	48.3	47.6	47.0	19.0	0.09	Ns
Weight, g	33.0	33.4	32.7	32.5	12.4	0.74	Ns
Diameter, mm	15.8	16.0	15.7	16.0	9.8	0.85	Ns
Length, mm	121.7	119.7	122.3	120.8	4.7	0.09	Ns
Seedor Index	271.3	279.04	267.9	269.1	11.8	0.29	Ns

¹ Control diet (CD): 3000 IU vitamin D_3_; ² CD + 2760 IU/kg 25(OH)D_3_ fed from 0 to 21 days; ³ CD + 2760 IU/kg 25(OH)D_3_ fed from 0 to 35 days; ^4^ CD + 2760 IU/kg 25(OH)D_3_ from 0 to 46 days; CV: coefficient of variation; Ns: not significant. (*n* = 54 birds/treatment; *p* < 0.05).

## Data Availability

Not applicable.
